# Research and application of intelligent diagnosis and treatment engineering bacteria

**DOI:** 10.3389/fbioe.2024.1524376

**Published:** 2024-12-18

**Authors:** Na Zhao, Junwei Chen, Jingtian Shi, Yan Gao, Lijing Li, Liyun Dong

**Affiliations:** ^1^ Department of Gynecology, Peking University First Hospital Ningxia Women and Children’s Hospital, Yinchuan, Ningxia, China; ^2^ School of Health Science and Engineering, University of Shanghai for Science and Technology, Shanghai, China; ^3^ Department of Pharmacy, Peking University First Hospital Ningxia Women and Children’s Hospital, Yinchuan, Ningxia, China; ^4^ Department of Gynecology and Obstetrics, Yinchuan Second People’s Hospital, Yinchuan, Ningxia, China

**Keywords:** intelligent diagnosis and treatment, genetic engineering, engineering bacteria, biosensor system, synthetic biology

## Abstract

For over a century, scientists have been harnessing the therapeutic potential of bacteria in treating diseases. The advent of synthetic biology in recent years has propelled the development of genetically engineered bacteria with enhanced intelligence. These bacteria can autonomously detect environmental cues and relay them to pivotal promoters, leading to the expression of functional proteins. By utilizing modular components, they are capable of executing a range of functions, including sensing, transmitting, and outputting signals. Based on these principles, a series of intelligent diagnostic and therapeutic engineered bacteria have emerged. These bacteria are capable of targeting diseased sites, sensing disease-specific signals, and producing reporter and therapeutic drugs. Furthermore, the integration of intelligent diagnostic and therapeutic engineered bacteria with advanced technologies such as artificial intelligence, nanomaterials, and optics has paved the way for diverse clinical applications. Three critical stages are explored in this article, which include the selection of strains, the design of biosensing systems, and the planning of release strategies. The application of intelligent diagnosis and treatment engineering bacteria in metabolic diseases, inflammatory diseases, tumors and infectious diseases is reviewed.

## 1 Introduction

The use of bacteria for treating diseases by humans dates back to the 19th century, when Dr. William Coley discovered that injecting patients with *Streptococcus pyogenes* and *Serratia marcescens* could lead to a reduction in tumor size. At the end of the 20th century, with the development of molecular biology and genetic engineering technology, scientists began to try to transform bacteria through genetic engineering to diagnose and treat diseases. Living engineered bacterial therapy emerged as the times require (Srivastava and Lesser, 2024). This therapy has the advantages of low cost, sensitivity and robustness, and can detect and treat diseases noninvasively and *in situ*, which is in line with the sustainable development goals of the World Health Organization (Tanniche and Behkam, 2023). In recent years, with the advancement of synthetic biology, genetically engineered bacteria have become increasingly intelligent, capable of sensing environmental signals and transmitting them to key promoters, ultimately leading to the expression of functional proteins (Zhang X. E. et al., 2023). The cell factors involved in the aforementioned processes are categorized into three modules based on their functions: the reception module (proteins that can sense specific chemical or physical signals inside or outside the cell and convert the signals to downstream modules); the transmission control module (one or a series of transcription factors and their target promoters); and the output module (proteins that can serve as markers or change the bacteria themselves and the surrounding environment) (Chen and Elowitz, 2021). These three modules constitute the core component of the intelligent engineered bacteria’s biosensing system (Brophy and Voigt, 2014; Gordley et al., 2016). Intelligent engineered bacteria have been applied in agriculture, energy, manufacturing, biology, and basic medical research (Smanski et al., 2016; Dobrin et al., 2016; Clarke and Kitney, 2020; Gao et al., 2019). This article reviews intelligent engineered bacteria used for disease diagnosis and treatment, introducing their general construction and application in disease research.

## 2 Construction of intelligent diagnosis and treatment engineering bacteria

Engineered bacteria for disease diagnosis and treatment are introduced into the host’s body through various routes such as oral administration (Steidler et al., 2003), intravenous injection (Ryan et al., 2009), enema (Ricci et al., 2003), vaginal administration (Liu et al., 2006), and intratumoral injection (Din et al., 2016). The environmental compatibility of the bacterial strains with the lesion site allows them to effectively reach the target location, moderately colonize at the lesion site, and sense specific physical and chemical signals in the environment. These signals are then transmitted into the engineered bacteria, where they are conducted through transcription factors and their corresponding promoters to controllably express diagnostic reporting factors or therapeutic factors. These factors can be effectively released from the engineered bacteria into the lesion site, thereby playing roles in real-time diagnosis, metabolic regulation, inflammation suppression, tumor cell killing, and anti-infection, and can be cleared from the host’s body after fulfilling their functions (Riglar and Silver, 2018). From the entire process, the selection of bacterial strains, the construction of the biosensing system, and the design of the release mechanism are three key aspects in constructing intelligent engineered bacteria for disease diagnosis and treatment.

### 2.1 Selection of bacterial strains

When selecting chassis strains for disease diagnosis and treatment, its biosafety and non pathogenicity should be considered first. In addition, genetic manipulation difficulties of chassis strains, that is, the adaptability to DNA transformation and the availability of genome manipulation tools, must also be considered. Finally, the survival and colonization ability of strains in the disease environment are also the key factors to achieve the purpose of diagnosis and treatment.

#### 2.1.1 Nonpathogenicity and biosafety

Nonpathogenicity are prerequisites for the selection of engineering bacteria chassis for diagnosis and treatment. Therefore, safe non pathogenic probiotics should be selected as chassis strains as far as possible. For example, *Escherichia coli* Nissle 1917 (EcN) is a gram negative probiotic that is sensitive to serum and does not produce enterotoxins or cytotoxins associated with pathogenic *Escherichia coli* (*Escherichia coli*) strains (Yu et al., 2023). However, the virulence of some chassis strains is inevitable, and the virulence of bacteria can be reduced by modifying the relevant genes of bacteria, so as to enhance its safety. For example, attenuated *Salmonella* strain ΔppGpp was engineered by regulating endotoxin gene expression. This strain is a double mutant (rela-, spot-), which has defects in ppGpp synthesis, resulting in downregulation of endotoxin gene expression (Na et al., 2006). Biosafety is a key factor in the approval of clinical trials and the use of engineered bacterial therapies. Preventing engineered bacteria from surviving after leaving the experimental and clinical environment is an important method to improve biosafety. Scientists have designed a killing switch in response to temperature changes. When the engineered bacteria leave the host, the cold induced promoter will express the toxin, thus reducing the survival rate of the strain (Stirling et al., 2017).

#### 2.1.2 Genetic manipulation difficulties

Gene modification technology plays a pivotal role in the in-depth exploration of biotechnology. The precise gene modification is essential for accurately regulating the functions of organisms and serves as an indispensable cornerstone in the construction of “intelligent” diagnostic engineering bacteria. Successful gene modification relies on reliable DNA transformation tools, effective control of gene expression, and post-translational control of protein processes, such as secretion (Arnold et al., 2023). In addition to these fundamental elements, the design of engineering bacteria also necessitates the integration of various advanced functional components, including sensors, circuit components, and actuators. *E*. *coli*, due to its clear genetic background, rapid propagation, low cost, and robust ability to express foreign proteins, has been employed as the primary strain for engineering development (Rosano et al., 2019). *Lactobacillus*, being easy to cultivate and cost-effective, possesses a variety of mature gene tools and vector systems, and can effectively target single membrane proteins of eukaryotic cells, making it a promising candidate chassis (Frelet-Barrand, 2022). Furthermore, thanks to the successful development of supportive genetic tools, including promoters, ribosome binding sites (RBS), vector backbones, and genome integration systems, the intestinal commensal bacterium *Bacteroides* polymorphus has also been gradually utilized as a new probiotic chassis (Lai et al., 2022).

#### 2.1.3 Survival and colonization ability

The survival and colonization capabilities of chassis strains at the lesion site are crucial for disease diagnosis and treatment. Taking ulcerative colitis, a challenging condition in gastroenterology, as an example, if the chassis strain can effectively colonize the ileocolonic region, it may provide significant assistance in the treatment of such diseases. Given that *Bacteroides* and other intestinal bacteria have a higher distribution in the colon, they can be considered as potential strains for the treatment of ulcerative colitis (Aitken et al., 2024). In contrast, since the pathological changes in Crohn’s disease can extend from the mouth to the anus, implying that the corresponding treatment strategies need to cover a broader intestinal area, the use of *Lactobacillus*, which can colonize both the small intestine and the colon, is likely to enhance the therapeutic effect on Crohn’s disease (Li et al., 2023). Furthermore, the nanoscale outer membrane vesicles (OMVs) produced by symbiotic gut bacteria *Bacteroides thetaiotaomicron* can penetrate the intestinal mucosa and directly interact with the innate layer immune cells, making it highly suitable as an engineered bacterial chassis for the diagnosis and treatment of intestinal diseases (Durant et al., 2020; Taketani et al., 2020).

### 2.2 Design of biosensing system

**FIGURE 1:**
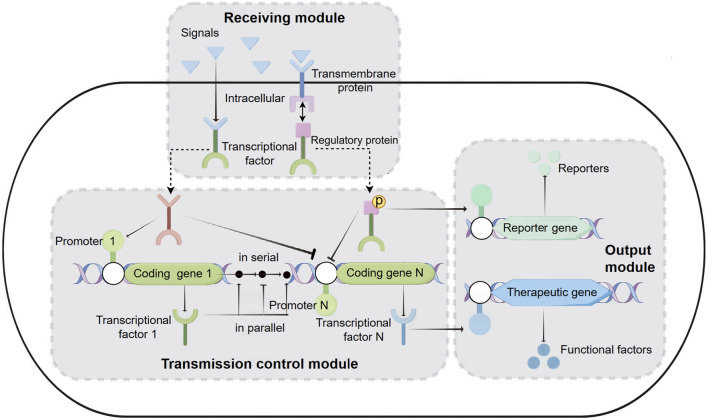
Biosensing System of Intelligent Diagnosis and Treatment Engineering Bacteria. Receiving module: intracellular reception: Physical and chemical signals from the disease environment enter the cell, interact with transcription factors, and cause conformational changes in transcription factors. Subsequently, transcription factors activate or inhibit inducible promoters on the transport control module or output module. Transmembrane reception: including a transmembrane protein and a transcriptional regulatory protein. Transmembrane proteins receive extracellular signals and phosphorylate transcription regulatory proteins. Then, transcriptional regulatory proteins bind to the promoters of transport or output modules, activating or inhibiting the transcription of coding genes. Transmission control module: located between the receiving module and the output module, it is composed of one or more transcription units connected in series or parallel. Output module: It can express reporter genes or therapeutic genes to achieve diagnostic and therapeutic purposes.

With the advancement of synthetic biology, biosensing systems are often integrated into the functional components of engineered bacteria (Gui et al., 2017; Ali et al., 2020). Therapeutic engineered bacteria have evolved from simply expressing therapeutic factors to becoming more intelligent, capable of first receiving disease signals (physical signals: temperature, pH; chemical signals: oxygen, ions, small molecule metabolites, peptides, sugars, *etc.*) and then transferring these signals to the transmission control module for the controllable output of therapeutic factors (Tabor et al., 2009). The entire biosensing system can be functionally divided into three modules: reception, transmission control, and output (Figure 1).

#### 2.2.1 Receiving module

Currently, the reception modules used in therapeutic engineered bacteria are divided into two types: intracellular reception modules and transmembrane reception modules (Figure 1). Intracellular reception modules typically refer to transcription factors that can sense signals within the cytoplasm of engineered bacteria. The physicochemical signals in the disease environment directly enter the cytoplasm, causing the transcription factors to undergo conformational changes. The conformationally changed transcription factors activate or inhibit the inducible promoters on the transmission control module or the output module, thereby controlling the expression of diagnostic reporting factors or therapeutic factors. For example, the zinc-responsive transcription factors Zur and ZntR can serve as reception modules that react with zinc ions entering the cell, and their integration into *E. coli* allows for the sensing of serum zinc levels and the production of visible pigments to identify zinc deficiency (McNerney et al., 2019). A protein, lasR, which reacts with acyl-homoserine lactone (AHL), has been used as an intracellular reception module in engineered *E. coli* to monitor the presence of pathogenic *Pseudomonas aeruginosa* (*P*.*aeruginosa*) in solid agar (Gupta et al., 2013), biofilms (Saeidi et al., 2011), and animal models (Hwang et al., 2017). Another transcriptional regulator, NorR, which is activated by nitric oxide (NO), has also become an intracellular reception module for engineered bacteria to detect inflammatory signals (Archer et al., 2012; Chen et al., 2021). Finally, various orally administered gut-adapted bacteria have been implanted with intracellular reception modules to sense lactose (Drouault et al., 2002), xylan (Hamady et al., 2010), and rhamnose (Mimee et al., 2015) in the diet, as well as chemical substances produced by the gut microbiota in mice (Pickard et al., 2014). Although intracellular reception modules play an important role in disease diagnosis, they also have limitations because they cannot sense extracellular disease signals.

Transmembrane reception modules are the primary means for engineered bacteria to sense extracellular stimuli (Gao and Stock, 2009; Galperin, 2010). The classic transmembrane reception module features a histidine kinase transmembrane protein that, upon stimulation by specific signals in the environment, phosphorylates histidine on the intracellular side of the membrane. It then phosphorylates a transcriptional regulatory protein, which can bind to the promoters of the transmission control module or the output module, activating or inhibiting the transcription of the encoded genes (Beier and Gross, 2006). Many transmembrane reception modules have been utilized for disease diagnosis and treatment (Lazar and Tabor, 2021). Two early-developed transmembrane reception modules from the marine bacterium *Shewanella*, thiosulfate sensor ThsS-ThsR and tetrathionate sensor TtrS-TtrR, respectively receive thiosulfate and tetrathionate signals across the membrane, and these modules have been designed to be installed in *E. coli* as a potential therapeutic means for intestinal inflammation (Daeffler et al., 2017; Riglar et al., 2017). Recently, researchers designed a NarX-NarL-based transmembrane reception module to detect nitrate levels, and in EcN, the combination of NarX-NarL with ThsS-ThsR modules across the membrane receives nitrate and thiosulfate, enhancing the specificity of the engineered bacteria in diagnosing intestinal inflammation (Woo et al., 2020). Another study integrated the transmembrane reception elements CqsS-LuxU-LuxO derived from *Vibrio cholerae* with a dCAS9-based green fluorescent protein (GFP) reporter gene system into *E. coli*, constructing a biosensor with high sensitivity to quorum sensing (QS) ligand cholera CAI-1, indicating the presence and proliferation of *Vibrio cholerae* (Holowko et al., 2016). The reception module can act directly on the output module or can be combined with the output module through the transmission control module to form a more intelligent biosensing system (Anderson et al., 2007).

#### 2.2.2 Transmission control module

The transmission control module is located between the reception module and the output module, and it is composed of several serial or parallel transcriptional units (Figure 1). The efficiency of the transmission control module is equal to the sum of the rates of all transcriptional units. The efficiency of each transcriptional unit depends on its promoter, terminator, and ribosome binding site (RBS) components. The promoter marks the starting point of transcription and can ensure the transcription rate of mRNA through a series of well-designed constitutive promoter libraries or chemically inducible promoters (Kelly et al., 2009; Mutalik et al., 2013; Yao et al., 2013; Lutz and Bujard, 1997; Cox et al., 2007; Armetta et al., 2021). Terminating the transcription process is crucial to avoid unintended interactions between different transcriptional units in the engineered DNA sequence. Previous studies have established numerous high-efficiency transcription terminator resource libraries, which include a large number of high-performing terminators (Chen et al., 2013; Cambray et al., 2013). The RBS determines the efficiency and location of ribosome binding, and it can regulate the rate of translation initiation, thereby controlling the expression level of proteins. By using RBS calculation tools to design specific ribosome binding sequences (Salis et al., 2009; Espah Borujeni et al., 2014; Farasat et al., 2014), or employing a more flexible “dual-cistron design” method with RBS (Mutalik et al., 2013), the translation efficiency of target genes in engineered bacteria can be conveniently adjusted.

**FIGURE 2:**
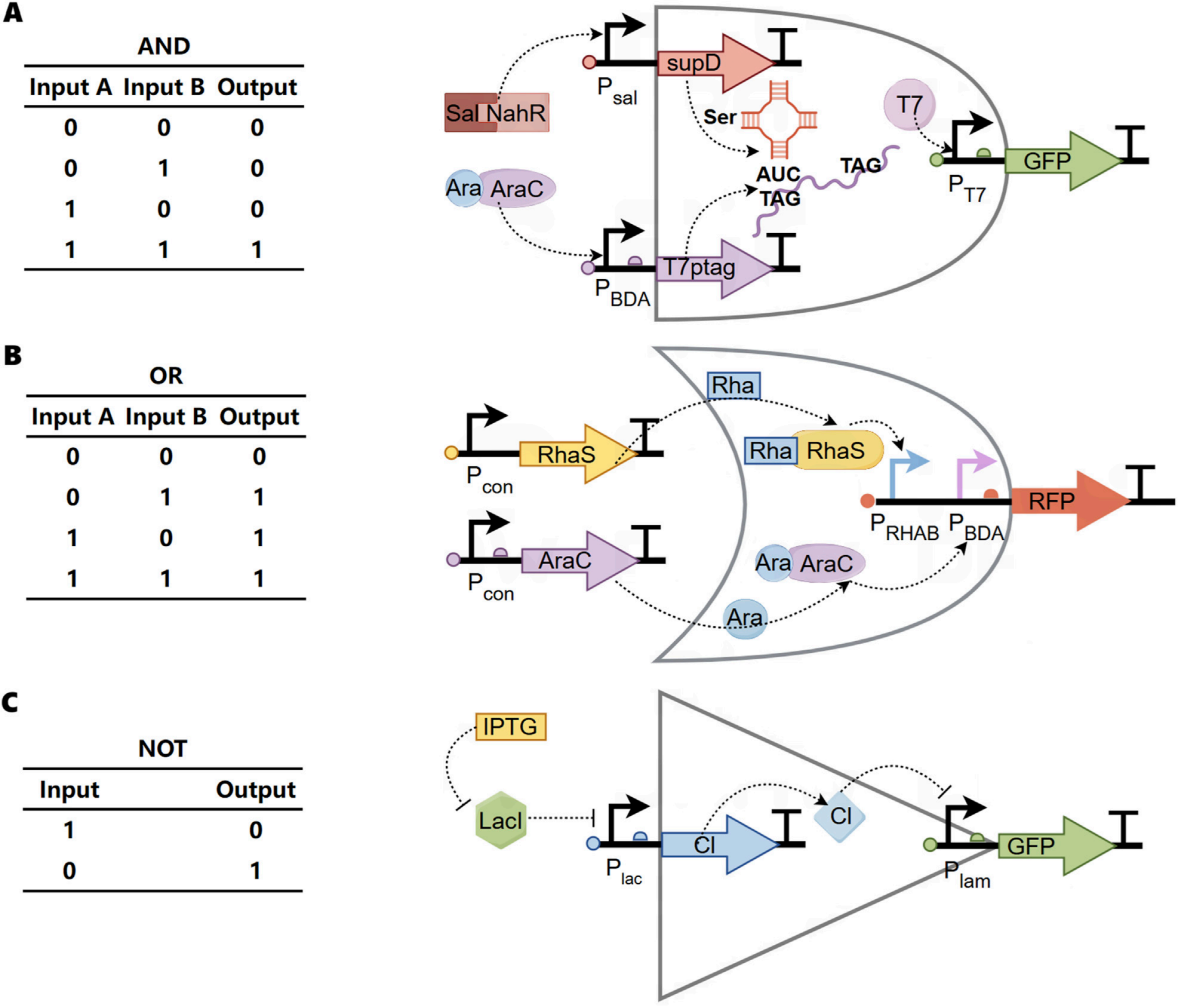
Boolean logic of transmission control module **(A)** Biological “AND gate”. Two input promoters were used that react with small molecule salicylates (Sal) and arabinose (Ara). The first promoter P_sal_ is related to the transcription of the amber inhibitory factor tRNA supD. The second promoter P_BAD_ drives transcription of T7 RNA polymerase. The polymerase gene has been modified to contain two amber stop codons (T7ptag). When supD is also transcribed, these stop codons are translated into serine. The polymerase T7 is only expressed in the presence of supd and T7ptag mRNA. Therefore, GFP can be output only when Sal and Ara are input at the same time. **(B)** Biological “OR gate”. P_BAD_ and P_RHAB_ promoters were designed upstream of an RFP reporter gene with strong RBS. When rhamnose (Rha) or arabinose (Ara) existed in the environment, they could combine with the corresponding receiving module, and then induce the expression of RFP. **(C)** Biological “NOT gate”. In the absence of isopropyl-β-d-thiogalactoside (IPTG), the repressor protein lacI will prevent the polymerase from binding to the promoter P_lac_ and cannot express CI. At this time, GFP can be expressed. When IPTG is present, IPTG inhibits lacI, CI repressor protein can be expressed, and then interacts with the promoter P_lam_ to inhibit the expression of GFP.

The transmission control module, based on Boolean logic, is divided into three basic modes: “AND gate transmission control” (functional factors are expressed only when both signals are present simultaneously) (Figure 2A) (Woo et al., 2020; Anderson et al., 2007), “OR gate transmission control” (functional factors are expressed as long as one of the two signals is present) (Figure 2B) (Wong et al., 2015), and “NOT gate transmission control” (functional factors are not expressed when a certain signal is present, otherwise they are expressed) (Figure 2C) (Bartoli et al., 2020). These three basic modes can also be combined to form advanced transmission control modules such as NAND (Wang et al., 2011), NOR (Tamsir et al., 2011), and XOR gates (Wong et al., 2015).

#### 2.2.3 Output module

The output module of intelligent therapeutic engineered bacteria is the functional gene that ultimately exerts its effect, which can be divided into two types: diagnosis and treatment (Figure 1).

Diagnostic output modules often express pigments, luciferases, or fluorescent proteins because the intensity of this form of output is easily observable and measurable. The advantage of pigments as diagnostic outputs is that they are visible to the naked eye without the need for special equipment. The earliest example is the enzymatic activity of the *lacZ* gene on X-gal, which produces a blue pigment (Horwitz et al., 1964). Violacein, lycopene, and carotenoids are induced to express by Zur and ZntR zinc-responsive transcription factors (TFs) to sense serum zinc levels for identifying zinc deficiency (McNerney et al., 2019). Luciferase, due to its very low background luminescence in fecal samples, typically produces higher sensitivity than pigment reporter genes. Although the use of specific equipment and substrates is required, these limitations do not hinder their significant role in improving the reliability of detection (Corthier et al., 1998). Fluorescent proteins are also commonly used as output proteins for disease diagnosis. Researchers have combined GFP and its variants (such as RFP, YFP, CFP) with transmembrane reception modules for the diagnosis of inflammatory bowel disease (IBD) (Daeffler et al., 2017; Riglar et al., 2017; Woo et al., 2020). This allows researchers to more accurately observe the morphology, growth status, and interactions of engineered bacteria at the single-cell level.

Therapeutic output modules express functional factors such as interleukins, hormones, enzymes, and bacterial toxins. *Lactobacillus* expressing interleukin-27 (IL-27) can be used to treat inflammatory bowel disease (Hanson et al., 2014). Interleukin-10 (IL-10) and proinsulin are co-expressed in *Lactobacillus* for the treatment of type 1 diabetes (Takiishi et al., 2017). Engineering EcN can bind to heparin sulfate proteoglycans (HSPG) on the surface of cancer cells and secrete a substance called myrosinase, which converts glucosinolates in food into sulforaphanes with anticancer activity (Ho et al., 2018). *E. coli* loaded with the bacterial toxin antimicrobial protein (S5 pyocin) and biofilm-degrading enzyme (DspB) has shown a synergistic effect in eliminating *P*. *aeruginosa*, improving the efficiency of pathogen clearance (Hwang et al., 2017).

### 2.3 Design of release method

**FIGURE 3:**
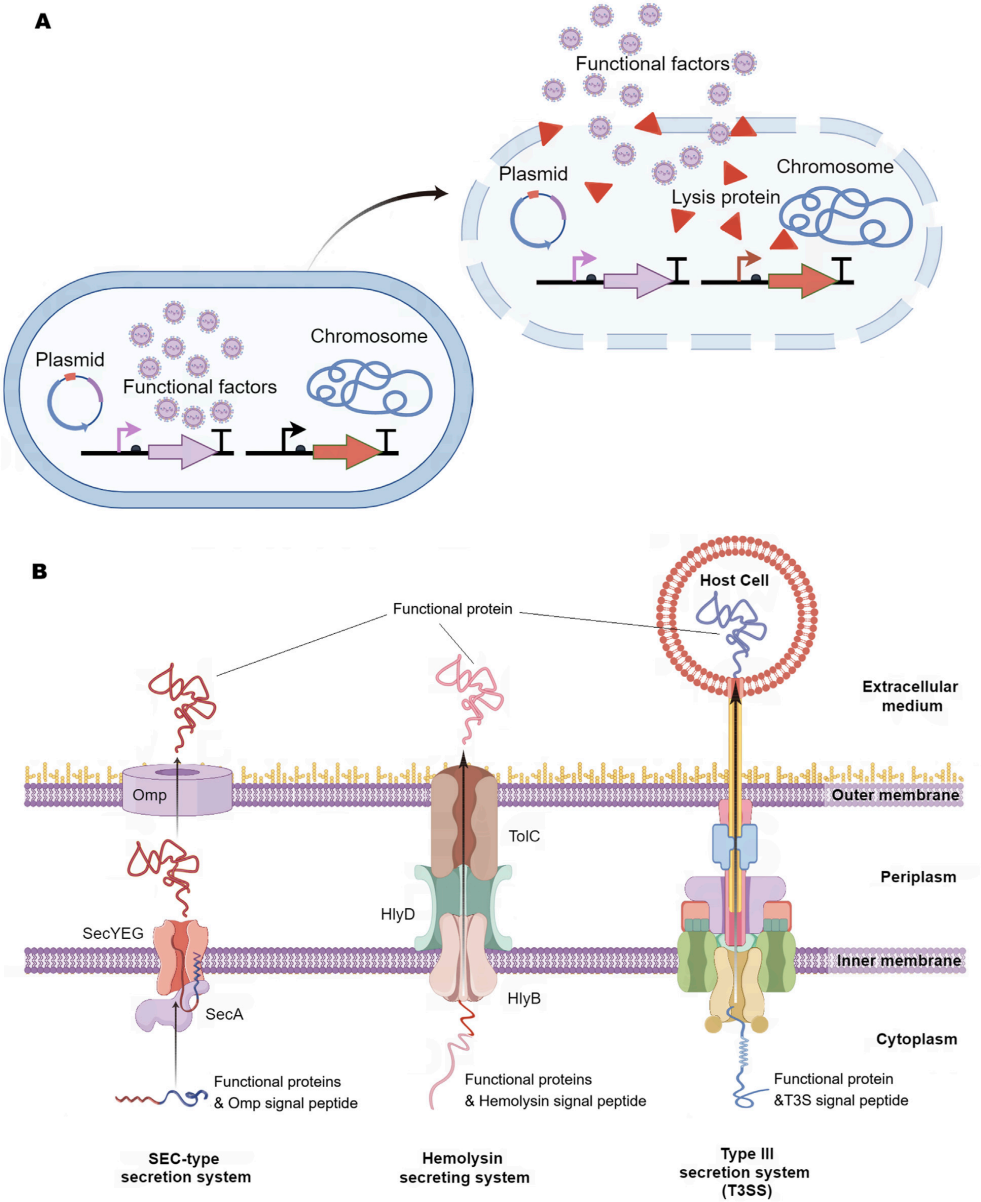
Release Method of Intelligent Diagnosis and Treatment Engineering Bacteria. **(A)** Bacterial lysis: Engineering bacteria first express functional factors, and when they reach the lesion, special signals in the disease environment induce engineering bacteria to express lytic proteins, followed by the release of bacterial lytic functional factors. **(B)** Extracellular secretion system: SEC-type secretion system, which first secretes functional proteins with signal peptides into the cytoplasm, and then secretes them out of the cell through outer membrane proteins. Hemolysin secretion system is comprised of three components (HlyB, HlyD, and TolC), which can secrete functional proteins carrying the HlyA signal peptide outside the cell. Type III secretion system (T3SS) can directly inject functional factors into host cells. Omp, outer membrane proteins; Hly, Hemolysin.

The release of functional factors from engineered bacteria into the lesion environment is essential for their pharmacological efficacy. These factors are typically released through mechanisms of bacterial lysis or via excretion systems (Figure 3). The cutting-edge lytic system derived from bacteriophage iEPS5 has been used to induce lysis of *Salmonella typhimurium*, promoting the release of mitochondrial targeted domain protein (MTD) (Jeong et al., 2014) and immunotoxin TGFα- PE38 (Lim et al., 2017) for cancer treatment (Figure 3A).

Among the various excretion systems, the SEC-type secretion system plays a pivotal role by linking a signal peptide to the functional factor, which aids in the translocation of the factor across the inner membrane of the engineered bacteria from the cytoplasm to the periplasm, ultimately secreting it into the surrounding environment (Zheng et al., 2017; Lim et al., 2017; Pöhlmann et al., 2012) (Figure 3B). The hemolysin secretion system is a continuous tunnel that spans the inner and outer membranes (composed of HlyB/HlyD/TolC), which can induce functional proteins with signal peptides to be excreted from the intracellular space (Gentschev et al., 2002; Zou et al., 2023) (Figure 3B). Researchers have implanted the Type III secretion system (T3SS) from pathogenic bacteria into non-pathogenic *E. coli*, which can directly inject functional factors into host cells (Reeves et al., 2015; Ruano-Gallego et al., 2015; González-Prieto and Lesser, 2018) (Figure 3B). Although these release systems have been proven to be effective in animal models, their safety for human disease applications still requires further validation.

## 3 Application of intelligent diagnosis and treatment engineering bacteria

Compared to other therapies, engineered bacteria can perform sensitive, noninvasive, and *in situ* detection and treatment of diseases at low cost (Tanniche and Behkam, 2023), and the rapid advancement of synthetic biology has further enhanced their stability and intelligence (Landry and Tabor, 2017). Consequently, the application of intelligent diagnostic and therapeutic engineered bacteria has been extended from gastrointestinal diseases to metabolic disorders, oncology, infectious diseases, and other areas (Lim and Song, 2019; Gurbatri et al., 2022).

### 3.1 Metabolic disorders

Metabolic disorders can lead to diseases such as diabetes and obesity. Most of these disease belongs to chronic diseases, and current treatment methods include long-term scheduled medication or injections. These therapies have significant side effects, high costs, and require patients to strictly follow the treatment plan. Consequently, the deployment of intelligent diagnostic and therapeutic engineered bacteria presents itself as a potential treatment strategy for metabolic diseases.

#### 3.1.1 Diabetes

Type 1 diabetes is an autoimmune disease that leads to insulin deficiency and ultimately the loss of function of pancreatic β-cells, necessitating lifelong treatment, particularly with long-term, scheduled insulin injections (Subramanian et al., 2024). Researchers have utilized *Lactobacillus gasseri* to express glucagon-like peptide 1 (GLP-1_7–37_) and engineered it to be released via a type III secretion system. Data from diabetic rats treated orally provide evidence for the use of engineered commensal bacteria in the treatment of diabetes (Duan et al., 2015). Oral administration of engineered *Lactococcus lactis* expressing both human proinsulin (PINS) and human interleukin-10 (hIL-10), in combination with low-dose systemic anti-CD3 monoclonal antibodies, can stably restore blood glucose levels in non-obese diabetic (NOD) mice (Takiishi et al., 2017; Takiishi et al., 2012). Similarly, engineered *Lactococcus lactis* co-expressing human glutamic acid decarboxylase (GAD65) and human interleukin-10 (hIL-10) has shown the same therapeutic efficacy (Robert et al., 2014).

Type 2 diabetes is widely concerned because of its incidence rate and complications (Tao et al., 2020). GLP-1 is an incretin hormone produced by intestinal cells that stimulates insulin secretion from the pancreas in a glucose-dependent manner. Exogenous GLP-1 analogs are used for the treatment of type 2 diabetes. *Lactobacillus lactis* (LL-pUBGLP-1) containing a plasmid encoding GLP-1 was used as a delivery system for GLP-1, and its effectiveness was demonstrated by the reduction in plasma glucose levels and increment insulin concentrations in obese type 2 diabetic rats (Zucker Diabetic Fatty rats, ZDF) following oral administration (Agarwal et al., 2014). Researchers have designed a GLP-1 mutant pentamer (5 × GLP-1), which is expressed and extracellularly anchored on the cell wall of lactic acid bacteria, and retains its *in vitro* bioactivity after being digested by intestinal trypsin into GLP-1 monomers (Lin et al., 2016). To induce a significant insulinotropic effect in diabetic animal models, further enhancement of the expression level of GLP-1 by lactic acid bacteria is required. Consequently, researchers have further mutated GLP-1 and linked it to form a GLP-1 hexamer (6 × mGLP-1), and the hexamer expressed by *E. coli* has been shown to exert a long-lasting hypoglycemic effect in type 2 diabetic mice (Xu et al., 2017). Additionally, exendin-4, a GLP-1 receptor agonist with a more prolonged bioactivity compared to GLP-1, has been effectively expressed and secreted by *Lactobacillus paracasei* transformed with a plasmid encoding the exendin-4 gene. The secreted exendin-4 significantly enhanced insulin secretion from INS-1 β-cells, providing a novel strategy for the treatment of diabetes with engineered bacteria (Zeng et al., 2016). Recently, researchers have orally administered optogenetic engineered *Lactococcus lactis* in the gut, which secretes GLP-1 to regulate metabolism under the control of a wearable optical device, and this strategy has effectively controlled blood glucose, body weight, and other characteristics in rat and mouse models (Zhang X. et al., 2023).

#### 3.1.2 Phenylketonuria

Phenylketonuria (PKU; also known as phenylalanine hydroxylase deficiency) is an autosomal recessive genetic disorder of phenylalanine (Phe) metabolism, where excessive Phe levels can lead to brain dysfunction (van Spronsen et al., 2021). To reduce Phe levels in the body, Synlogic Corporation has designed a strain, EcN (SYNB1618), capable of expressing genes for phenylalanine transport protein (PheP), phenylalanine ammonia lyase (PAL), and L-amino acid deaminase (LAAD), which can convert Phe to trans-cinnamic acid (TCA) and phenylacetate (PP) in the gastrointestinal tract, effectively reducing blood Phe levels in mice and crab-eating macaques (Isabella et al., 2018). Subsequently, researchers conducted human trials with EcN (SYNB1618), which demonstrated safety and good tolerability (Puurunen et al., 2021). Building upon the success of EcN (SYNB1618), the entire cell PAL activity was optimized, resulting in a more effective strain, EcN (SYNB 1934), which showed approximately twice the PAL activity *in vivo* compared to its predecessor (Adolfsen et al., 2021).

#### 3.1.3 Obesity

Obesity is a major global issue that significantly impacts public health (Perdomo et al., 2023). In recent years, researchers have also made attempts to treat obesity using smart engineered bacteria. N-acylphosphatidylethanolamines (NAPEs) synthesized in the small intestine can enhance satiety and reduce food intake. Engineered EcN, through genetic modification, can synthesize NAPEs in the gut, thereby treating obesity. This approach is more effective and enduring than traditional dietary and lifestyle interventions (Chen et al., 2014). Butyrate can reduce appetite and alleviate fatty liver through gut-brain neural circuits. Transgenic *Bacillus subtilis* SCK6 can increase butyrate production, which has beneficial effects on obesity, fasting blood glucose, insulin resistance, hepatic steatosis, and fat accumulations (Bai et al., 2020). Engineered *Lactobacillus* expressing GLP-1 has an ameliorative effect on obesity in mice induced by a high-fat diet (HFD) (Wang et al., 2021).

#### 3.1.4 Hyperammonemia

Ammonia (NH_3_) is a toxic metabolite, which is produced in the small intestine and colon. The main route of ammonia (NH_3_) is to synthesize urea in the liver and excrete it in the urine from the kidney. Hyperammonemia occurs when there is a defect in ammonia metabolism. A large amount of ammonia that is not metabolized into urea by the liver enters the brain through the blood circulation, which can lead to brain injury and death (Ribas et al., 2022). Kurtz et al. Modified the metabolism of probiotic ECN, deleted the gene encoding arginine repressor ArgR, and then integrated the gene *Arga215* encoding ArgAY19C into the intergenic region of *malE* and *malK* genes under the control of endogenous anaerobic inducible promoter *fnrS* (P_*fnrs*_) of *Escherichia coli*. ArgAY19C is the feedback resistant version of N-acetylglutamate synthase ArgA *(argA* ^*fbr*^). This engineered strain, SYNB1020, can overproduce arginine, thereby sequestering some ammonia produced by intestinal bacteria into amino acid molecules. The experiment proved that synb1020 could reduce blood ammonia, improve the survival rate of mouse hyperammonemia model, and showed repeated dose tolerance in non-human primates. A phase 1 dose escalation study in healthy human volunteers did not result in serious adverse events and showed that the bacterium was metabolically active *in vivo*, indicating that SYNB1020 needs further clinical development (Kurtz et al., 2019). Subsequently, the researchers conducted experiments using two engineered EcN strains, S-ARG (SYNB1020) and S-ARG + BUT (SYNB1536), in a rat model of chronic liver injury (CLD) and hepatic encephalopathy induced by bile duct ligation (BDL). The S-ARG (SYNB1020) strain showed positive benefits in reducing hyperammonemia, thereby alleviating memory impairment in BDL rats. The S-ARG + BUT (SYNB1536) strain exhibits similar therapeutic effects in hyperammonemia and shows additional positive effects in systemic inflammation and endotoxemia, leading to adequate protection against memory deterioration (Ochoa-Sanchez et al., 2021).

#### 3.1.5 Hyperuricemia and gout

Uric acid is the final product of purine degradation in the human body, which can be excreted from the body through the kidneys and intestines. Excess uric acid will lead to hyperuricemia. The chronic form of arthritis caused by precipitation of sodium urate crystals is called gout. Gout afflicts hundreds of millions of people worldwide, with a high incidence rate in developed countries (Borghi et al., 2020). Yang et al. discovered a new method to eliminate purines from the diet by engineering gut bacteria, integrating a set of enzymes and transporters capable of degrading purines under anaerobic conditions into the EcN chromosome, which can bypass the formation of uric acid. By orally supplementing these engineered probiotics or purified recombinant enzymes, it is possible to effectively treat hyperuricemia fruit fly models, providing a new approach for developing novel probiotic therapies for the treatment of hyperuricemia and gout (Tong et al., 2023). Zou et al. also developed a probiotic EcN called YES301, which significantly improved the ability of bacteria to uptake xanthine and hypoxanthine by rationally designing overexpression of the xanthine transporter XanQ. As a result, serum uric acid levels were reduced and kidney damage was improved in a hyperuricemia mouse model. YES301 has shown comparable efficacy to traditional uric acid lowering drugs such as allopurinol, but with fewer side effects and higher biosafety, highlighting the potential of engineered probiotics in reducing intestinal purine levels to manage hyperuricemia (Zou et al., 2024).

### 3.2 Inflammatory bowel disease

Inflammatory bowel disease (IBD), which includes Crohn’s disease and ulcerative colitis (UC), is a type of incurable inflammatory condition (McDowell et al., 2024; Lu et al., 2022; Taku et al., 2020). The oral administration of smart diagnostic and therapeutic engineered bacteria, equipped with an internal biosensing system that can directly deliver therapeutic molecules to the gut, offers a highly promising approach for the diagnosis and treatment of inflammatory bowel disease (IBD).

In the early diagnosis of IBD, Archer et al. (2012) used the transcriptional regulator NorR as a receiving module that reacts with nitric oxide (NO) released during intestinal inflammation, thereby relieving the repression of the promoter and activating the expression of the DNA recombinase FilmE. FilmE can reverse the expression type of the fluorescent protein, from yellow fluorescent protein (YFP) to cyan fluorescent protein (CFP), and the change in fluorescence color can serve as a basis for the diagnosis of intestinal inflammation. Another study designed an *E. coli* strain (NGF1) that uses the transmembrane receiving module TtrS-TtrR to sense tetrathionate in the inflammatory intestinal environment, then remembers the presence of tetrathionate through the phage CI/Cro control module, and finally expresses β-galactosidase (β-gal) as an output. The β-gal in the feces reacts with a reagent to produce a color change, which can serve as a diagnostic basis for intestinal inflammation. This smart diagnostic engineered bacterium is effective for a long period within mice (Riglar et al., 2017).

Engineered bacterial strains that directly or indirectly express anti-inflammatory factors to inhibit pro-inflammatory factors show great promise in the treatment of IBD. Cui et al. constructed a photosensitive *E. coli* strain that uses YF1/FixJ as a transmembrane receiving module, capable of sensing blue light to secrete IL-10, and this system demonstrated real-time monitoring and long-term alleviation effects in acute and chronic UC mouse models (Cui et al., 2021). Recently, Zou et al. designed an EcN named i-ROBOT for real-time diagnosis and treatment of IBD. The transmembrane receiving module thsS/thsR of i-ROBOT senses the inflammatory signal thiosulfate, triggering the expression of the diagnostic factor superfolder green fluorescent protein (sfGFP) and the therapeutic factor immunomodulator AvCystatin. The therapeutic factor AvCystatin, connected with the Hly signal peptide, is released into the lesion through the implanted hemolysin secretion system to exert its pharmacological effect. At the same time, the inserted codon ACG in the chromosome is edited by BE2 and sgRNA to become the start codon ATG, initiating the expression of LacZ, which serves as a signal recording function. DSS-induced colitis in mice treated with i-ROBOT by gavage effectively alleviated the disease (Zou et al., 2023). Chua et al. designed an EcN strain using the endogenous regulatory factor NorR as a receiving module to respond to the colorectal inflammatory marker nitric oxide (NO), then activating the inducible promoter pNorV to express interferon lambda 1 (IFNL1). IFNL1 is excreted from the bacteria with the help of the signal peptide YebF, acting on immune cells to regulate inflammatory pathways. This engineered bacterium played an anti-inflammatory role in two *in vitro* IBD models (Chua et al., 2023). Researchers administered *Lactobacillus* expressing the antimicrobial peptide (LL37) to mice by continuous gavage for 10 days, with a single rectal injection of the inflammatory inducer DNBS on the 7th day, and the results proved that this engineered bacterium could prevent the occurrence of colitis and alleviate colitis symptoms (Noguès et al., 2022). In addition, *Lactobacillus* has been engineered to express IL-10 (Steidler et al., 2000), IL-27 (Hanson et al., 2014), IL-35 (Wang et al., 2019), thymic stromal lymphopoietin (TSL) (Aubry et al., 2015), and heme oxygenase (PHO-1) (Shigemori et al., 2015), among other immune factors, for the treatment of IBD.

Restoring intestinal mucosal healing and epithelial integrity is also a strategy for the treatment of IBD. Liang et al. (2022) directly inserted the nattokinase gene, connected with a signal peptide, into the natural plasmid pMUT1 of EcN. As a therapeutic factor, nattokinase alleviates DSS-induced intestinal epithelial injury and restores mucosal integrity by upregulating the levels of tight junction proteins (including ZO-1 and occludin). Praveschotinunt et al. (2019) linked the trefoil factor family (TFF) with a membrane protein (CsgA) to form a mucosal component that can protect the intestinal epithelium. This component, expressed by EcN and secreted onto the intestinal mucosa through the curli secretion system, can promote the repair of DSS-induced intestinal epithelial cells in mice.

The production of reactive oxygen species (ROS) leads to oxidative stress, which can damage intestinal cells. Increasing the activity of enzymes that can scavenge ROS becomes another strategy for the treatment of IBD (Montero-Blay et al., 2023). Zhou et al. (2022) engineered EcN to express superoxide dismutase (SOD) and catalase (CAT), which are capable of scavenging ROS. In a mouse model of IBD, this engineered bacterium, coated with chitosan/alginate, effectively alleviated colonic inflammation and unexpectedly increased the abundance of important microorganisms that maintain intestinal homeostasis.

### 3.3 Cancer

The use of engineered bacteria for cancer therapy is an emerging biotherapeutic approach that leverages genetically modified bacteria to combat cancer cells. This method primarily operates through the following three mechanisms: releasing natural toxins to induce apoptosis in cancer cells, activating the immune system to recognize and eliminate cancer cells, and delivering anticancer drugs directly to the tumor site (Weerakkody and Witharana, 2019; Li et al., 2021; Yang et al., 2021).

#### 3.3.1 Releasing natural toxins promotes cancer cell apoptosis

Firstly, many bacteria contain natural toxins with potential anti-cancer properties. To better utilize these bacterial toxins for cancer treatment, scientists have recombined the natural toxin genes to express and purify them through engineered bacteria. This not only enhances the targeting of cancer cells but also reduces damage to normal tissues. For instance, researchers have mutated the *P*. *aeruginosa exotoxin* A (PE) and fused it with anti-CD25 to form a new recombinant toxin (CD25-PE38KDELKQK). The recombinant toxin, expressed and purified by *E*. *coli*, significantly reduced the natural toxin’s toxicity to the pulmonary vessels in the lungs of mice with lung cancer (Wang et al., 2008). Researchers have also linked the B subunit of the natural bacterial toxin Shiga-like toxin (STXB) with the DT389 fragment of the natural diphtheria toxin (DT538) to form a fusion toxic protein (DT389-STXB). STXB is responsible for recognizing the cancer cell surface receptor GB3, while the DT389 toxin is responsible for eliminating cancer cells, showing high affinity for breast cancer cells (Mohseni et al., 2021).

#### 3.3.2 Activate the immune system to recognize and eliminate cancer cells

Engineering bacteria can activate immune cells or guide them to recognize and kill cancer cells by expressing specific immune-stimulating molecules, such as cytokines or chemokines. This approach enhances the immune memory effect, preventing the recurrence of cancer.

Interleukin-2 (IL-2) can activate immune cells infiltrating tumor tissues, including T cells, NK cells, and ILC cells (Raeber et al., 2022). By electroporation of plasmids carrying the human IL-2 gene into the attenuated *Salmonella typhimurium* strain x455O, a drug delivery system x455O (pIL2) is constructed. Gavage of this engineered bacteria in MC-38 adenocarcinoma mice not only reduced liver metastasis of MC-38 adenocarcinoma but also did not observe toxicity of IL-2 or *Salmonella* (Saltzman et al., 1996). Subsequently, researchers successively attempted to use *Clostridium butyricum* secreting rat interleukin-2 (rIL2) (Barbé et al., 2005), and *Bacillus cereus* secreting mouse interleukin-2 (mIL2) as engineered bacteria for tumor treatment (Kubiak et al., 2021). Recently, scientists have progressively optimized the bioactivity of IL-2 using EcN, and mice with colon cancer models treated with intravenous injection of this engineered bacterial strain showed a moderate reduction in tumor growth rate and a significant increase in IL-2 levels in the tumor (Tumas et al., 2023).

Components of the interleukin-18 (IL-18) signaling pathway are upregulated on tumor-infiltrating lymphocytes, indicating that IL-18 can enhance the immune system’s ability to combat tumors (Zhou et al., 2020). Researchers synthesized IL-18 using an engineered attenuated *Salmonella typhimurium* strain (VNP20009), and after intravenous injection of this engineered bacteria into a mouse model, the growth of colon cancer and breast cancer cells was significantly inhibited, as well as lung metastasis in immunocompetent mice (Loeffler et al., 2008).

L-arginine in tumor tissue can enhance the antitumor capability of immune checkpoint inhibitors. To increase the production of L-arginine, scientists deleted the arginine repressor gene (*ArgR*) of EcN and mutated the N-acetylglutamate synthase gene (*ArgAfbr*). The engineered bacteria, when co-injected with anti-PD-L1, significantly reduced tumor growth rate (Canale et al., 2021).

Researchers used EcN with the lysis gene (*ΦX174E*) to express nanobodies targeting immune checkpoints PD-L1nb and CTLA-4nb. After intratumoral injection of this engineered bacteria, the tumor partially or completely regressed in the mouse model, and no visible liver metastasis was observed (Gurbatri et al., 2020).

Another study utilized engineered *Salmonella* (△ppGpp) to secrete the Del-1 protein (a potent inhibitor of neutrophil recruitment, antagonizing the antigen-1 associated with lymphocyte function on vascular endothelial cells). After tail vein injection of this engineered bacteria into a mouse model, the number of neutrophils in the tumor decreased, the number of M1-type macrophages increased, and tumor elimination was observed in some samples (Tian et al., 2022).

#### 3.3.3 Transporting anti-cancer drugs directly to the tumor microenvironment

Engineering bacteria can serve as drug delivery systems, directly delivering anticancer drugs to tumor cells. This approach can increase the local concentration of drugs and reduce systemic side effects. Researchers have used *E*. *coli* DH5α that can specifically target tumor tissues to express beta-glucuronidase (βG), which converts inactive prodrugs into anticancer active drugs at the tumor site, significantly inhibiting tumor growth (Cheng et al., 2008; Afkhami-Poostchi et al., 2020).

Bacterial spores can also serve as carriers for anticancer drugs because obligate anaerobic bacterial spores can only germinate in the hypoxic necrotic areas present in solid tumors, while remaining dormant in other parts of the body (Barbé et al., 2006; Kubiak and Minton, 2015). However, the original bacterial spores cannot eliminate cancer cells and require genetic modification or combination with other anticancer strategies. Scientists have covalently attached the chemotherapeutic drug gemcitabine (MGEM) to *Clostridium butyricum* spores (SPORE), and after oral administration, the drug (SPORE-MGEM) migrates from the upper gastrointestinal tract to pancreatic tumors, with a threefold increase in drug accumulation within the tumor compared to MGEM alone. In a mouse model of pancreatic cancer, SPORE-MGEM inhibited tumor growth without significant side effects (Han et al., 2022).

### 3.4 Infectious diseases

#### 3.4.1 AIDS

The primary mode of transmission for HIV/AIDS is sexual contact, with the cervicovaginal mucosa being the main route of HIV infection in women. Scientists have engineered *Lactobacillus jensenii*, isolated from vaginal mucosa, to express a bispecific CD4 (2D CD4) molecule on its surface that can bind to HIV proteins, which can impede heterosexual transmission of HIV (Chang et al., 2003; Liu et al., 2008). Subsequently, a study used engineered *Lactobacillus acidophilus* (ATCC 4356) to express human CD4, with the expressed CD4 protein anchored on the outer wall of the engineered bacteria to adsorb HIV-1 virus. In a humanized mouse model, the engineered bacteria, when stimulated rectally, prevented HIV-1 infection, but the vaginal effect was suboptimal (Wei et al., 2019). Similarly, the probiotic EcN was designed to secrete HIV-gp41-lysin peptides, thereby preventing HIV fusion and subsequent invasion. This engineered bacterial strain can colonize persistently and stably in the colon and cecum, releasing anti-HIV peptides in mice for several months (Rao et al., 2005).

#### 3.4.2 Cholera

Cholera is a life-threatening gastrointestinal infectious disease caused by the infection of toxigenic *Vibrio cholerae* (Kanungo et al., 2022). Engineered bacterial interventions have been used for the diagnosis and treatment of cholera. Scientists have constructed an intelligent engineered bacterium using *Lactococcus lactis*, whose recombinant transmembrane receptor module HR4M can specifically detect the quorum-sensing signal CAI-1 of *Vibrio cholerae* in the intestine and trigger the expression of the reporter gene β-lactamase. The expressed product is easily detectable in fecal samples, thereby assisting in the diagnosis of cholera (Mao et al., 2018). Holowko et al. (2016) developed an intelligent diagnostic engineered bacterium based on the quorum-sensing mechanism of *Vibrio cholerae* and CRISPRi technology for detecting *Vibrio cholerae*. It uses *E*. *coli* as a vector, and when *Vibrio cholerae* is at a high density, the transmembrane receptor module CqsS-LuxU-LuxO reacts with the signal molecule CAI-1, and the CRISPRi-based transmission control system triggers the expression of the diagnostic factor GFP, which has been successfully used for detecting *Vibrio cholerae*. Similarly, Jayaraman et al. (2017) replaced the GFP from the previous study with the lytic protein YebF-Art-085, and introduced the expression of the killing protein Art-085. The lysed engineered bacteria release the killing protein, thereby eliminating *Vibrio cholerae*. *In vitro* experiments have shown that this intelligent engineered bacterium can effectively inhibit the growth of *Vibrio cholerae* cells.

#### 3.4.3 *Pseudomonas aeruginosa* infection

*P*.*aeruginosa* primarily colonizes the respiratory and gastrointestinal tracts (Ungor and Apidianakis, 2024). It is one of the main causes of hospital-acquired infections due to its resistance to many antibiotics and antimicrobial agents (Jurado-Martín et al., 2021). Utilizing engineered bacteria to effectively sense and kill *P*. *aeruginosa* provides a novel antibacterial strategy for controlling infections caused by this bacterium. Researchers have designed a smart *E*. *coli* that can detect acyl-homoserine lactone 3OC12HSL produced by *P*. *aeruginosa* using the intracellular receptor protein lasR. Upon detection, the bacteriocin Pyocin S5 is produced, which can be released by the E7 lysis protein after lysing *E*. *coli*, and then kills *P*. *aeruginosa* in the infected environment (Saeidi et al., 2011). Building on the aforementioned research, another study changed the release mode of the smart engineered bacteria from lysis to secretion. When lasR binds to the *P*. *aeruginosa* infection signal 3OC12HSL, the pathogen-specific toxin CoPy, which carries the signal peptide Flgm, is expressed and can be released through the flagellar secretion system. This design can obviously kill *P*. *aeruginosa* more effectively and for a longer duration (Gupta et al., 2013). Subsequently, a similar study used the probiotic strain EcN as a host and added the expression of the biofilm-degrading enzyme DspB to the bactericidal protein Pyocin S5. This engineered bacterium showed both preventive and therapeutic activity in *Caenorhabditis elegans* and mouse models of *P*. *aeruginosa* intestinal infection (Hwang et al., 2017). Recently, Gao et al. (2023) constructed a chimeric pyocin (ChPy) to specifically kill *P*. *aeruginosa* and designed a near-infrared (NIR) light-responsive strain to produce and deliver this drug. This smart engineered bacterial strain can continuously produce ChPy without light and release it to kill *P*. *aeruginosa* upon NIR light-induced bacterial lysis.

## 4 Discussion

Although intelligent engineered bacteria have achieved preliminary research results in disease diagnosis and treatment, there are still a series of challenges and issues to be faced before they can be applied clinically. First, most of the research on therapeutic engineered bacteria is still focused on *in vitro* testing and animal experiments. While these experiments are important, they do not fully simulate the human body’s environment. Therefore, more rigorous clinical trials are needed to prove the actual efficacy and safety of therapeutic engineered bacteria. Second, the biosensing systems of the diagnostic and therapeutic engineered bacteria that have been developed are relatively simple, with some having only a single input and output, and some only containing basic logic control, which is not intelligent enough. Faced with complex disease environments, it is necessary to design biosensing systems with signal storage, automatic calibration, and self-learning capabilities to improve the specificity and targeting of intelligent diagnostic and therapeutic engineered bacteria. Finally, the ethics, legal issues, and social acceptance of intelligent diagnostic and therapeutic engineered bacteria are also issues that cannot be ignored. How to balance innovation and risk, ensure that technological development complies with ethical standards, and gain public understanding and support are necessary conditions for promoting the development of intelligent diagnostic and therapeutic engineered bacteria.

Despite the many challenges, with the comprehensive progress and in-depth research in various fields such as synthetic biology, artificial intelligence, and nanomaterials, interdisciplinary cooperation has injected new vitality and hope into the development of intelligent diagnostic and therapeutic engineered bacteria. The rapidly developing field of synthetic biology continuously provides various components for intelligent diagnostic and therapeutic engineered bacteria. These include easily manipulated engineered bacterial strains, biosensing systems with computational capabilities, storage capacity, and calibration functions. These standardized module libraries make the development of intelligent diagnostic and therapeutic engineered bacteria more convenient (Zhang C. et al., 2023). With the integration of artificial intelligence and big data technology in the field of bioinformatics, researchers have built numerous computational platforms for predicting protein structures and simulating drug-target interactions (Gupta et al., 2021). These platforms have greatly accelerated the research and development and evolution of intelligent diagnostic and therapeutic engineered bacteria. Innovations in fields such as optics (Gao et al., 2023), ultrasound technology (Bourdeau et al., 2018; Lakshmanan et al., 2020), and nanomaterials (Cao et al., 2023; Ma et al., 2023) have opened up multiple paths for the widespread clinical application of intelligent diagnostic and therapeutic engineered bacteria.

In summary, intelligent diagnostic and therapeutic engineered bacteria will play an important role in the future medical field. It will open up a new method of treatment and inject new vitality into the cause of human health.
